# Assessing the safety of lipid-modifying medications among Chinese adolescents: a drug-target Mendelian randomization study

**DOI:** 10.1186/s12916-023-03115-y

**Published:** 2023-10-31

**Authors:** Shan Luo, Hugh Simon Lam, Yap Hang Chan, Clara Sze Man Tang, Baoting He, Man Ki Kwok, Gabriel M. Leung, C Mary Schooling, Shiu Lun Au Yeung

**Affiliations:** 1 School of Public Health, Li Ka Shing Faculty of Medicine, The University of Hong Kong, 1/F, Patrick Manson Building, 7 Sassoon Road, Hong Kong SAR, China; 2 Department of Paediatrics, Faculty of Medicine, The Chinese University of Hong Kong, Shatin, Hong Kong SAR, China; 3 Division of Cardiology, Queen Mary Hospital, The University of Hong Kong, Hong Kong SAR, China; 4 Department of Surgery, Li Ka Shing Faculty of Medicine, The University of Hong Kong, Hong Kong SAR, China; 5 Dr. Li Dak-Sum Research Centre, The University of Hong Kong, Hong Kong SAR, China; 6 School of Public Health and Health Policy, City University of New York, New York, NY USA

**Keywords:** HMGCR inhibitors, PCSK9 inhibitors, Safety, Adolescents, Drug-target Mendelian randomization

## Abstract

**Background:**

With increasing hypercholesterolemia prevalence in East Asian adolescents, pharmacologic interventions (e.g., HMGCR inhibitors (statins) and PCSK9 inhibitors) may have to be considered although their longer-term safety in the general adolescent population is unclear. This study aims to investigate the longer-term safety of HMGCR inhibitors and PCSK9 inhibitors among East Asian adolescents using genetics.

**Methods:**

A drug-target Mendelian randomization study leveraging the Global Lipid Genetics Consortium (East Asian, *n* = 146,492) and individual-level data from Chinese participants in the Biobank clinical follow-up of Hong Kong’s “Children of 1997” birth cohort (*n* = 3443, aged ~ 17.6 years). Safety outcomes (*n* = 100) included anthropometric and hematological traits, renal, liver, lung function, and other nuclear magnetic resonance metabolomics. Positive control outcomes were cholesterol markers from the “Children of 1997” birth cohort and coronary artery disease from Biobank Japan.

**Results:**

Genetic inhibition of HMGCR and PCSK9 were associated with reduction in cholesterol-related NMR metabolomics, e.g., apolipoprotein B (HMGCR: beta [95% CI], − 1.06 [− 1.52 to − 0.60]; PCSK9: − 0.93 [− 1.56 to − 0.31]) and had the expected effect on the positive control outcomes. After correcting for multiple comparisons (*p*-value < 0.006), genetic inhibition of HMGCR was associated with lower linoleic acid − 0.79 [− 1.25 to − 0.35]. Genetic inhibition of PCSK9 was not associated with the safety outcomes assessed.

**Conclusions:**

Statins and PCSK9 inhibitors in East Asian adolescents appeared to be safe based on the outcomes concerned. Larger studies were warranted to verify these findings. This study serves as a proof of principle study to inform the medication safety among adolescents via genetics.

**Supplementary Information:**

The online version contains supplementary material available at 10.1186/s12916-023-03115-y.

## Background

Three-hydroxy-3-methylglutaryl coenzyme A (HMGCR) reductase inhibitors, collectively referred to as statins, are the first-line pharmacologic treatments for children with familial hypercholesterolemia (FH), which demonstrated a reduction in cardiovascular risk in adulthood [1]. However, with increasing rates of obesity in the adolescent populations, in particular East Asians [2], prescriptions of lipid-modifying medications may have to be considered for non-FH adolescents who have elevated, uncontrolled lipid profiles to mitigate the subsequent risk of atherosclerotic cardiovascular disease. Unfortunately, a recent review commented a lack of safety studies on these medications in adolescents [3]. As such, it would be of public health interest to evaluate any possible side effects of the use of these medications in the general adolescent population [4].

Real-world data, such as pharmaco-epidemiologic studies of medication side effects, are susceptible to confounding by indication or immortal time bias [5]. The use of drug-target Mendelian randomization studies has gained popularity in recent years, although only a few studies have been conducted to study the side effects of medications in European adult populations, such as anti-hypercholesteremia and antihypertensives [6–9]. The extent to which these side effects can be extrapolated to the adolescent population remains unclear. To better evaluate possible safety issues associated with statin use in Chinese adolescents, who constitute a significant proportion of the global population, we evaluated the association of genetically proxied inhibition of HMGCR with a wide range of phenotypes (*n* = 100) in an originally population-representative birth cohort in Hong Kong. For completeness, we also considered genetic inhibition of proprotein convertase subtilisin/kexin type 9 (PCSK9) and Niemann-Pick C1-Like 1 (NPC1L1) as these are emerging classes of medications for the treatment of hypercholesteremia in adolescents [10].

## Methods

### Study design

**Fig. 1:**
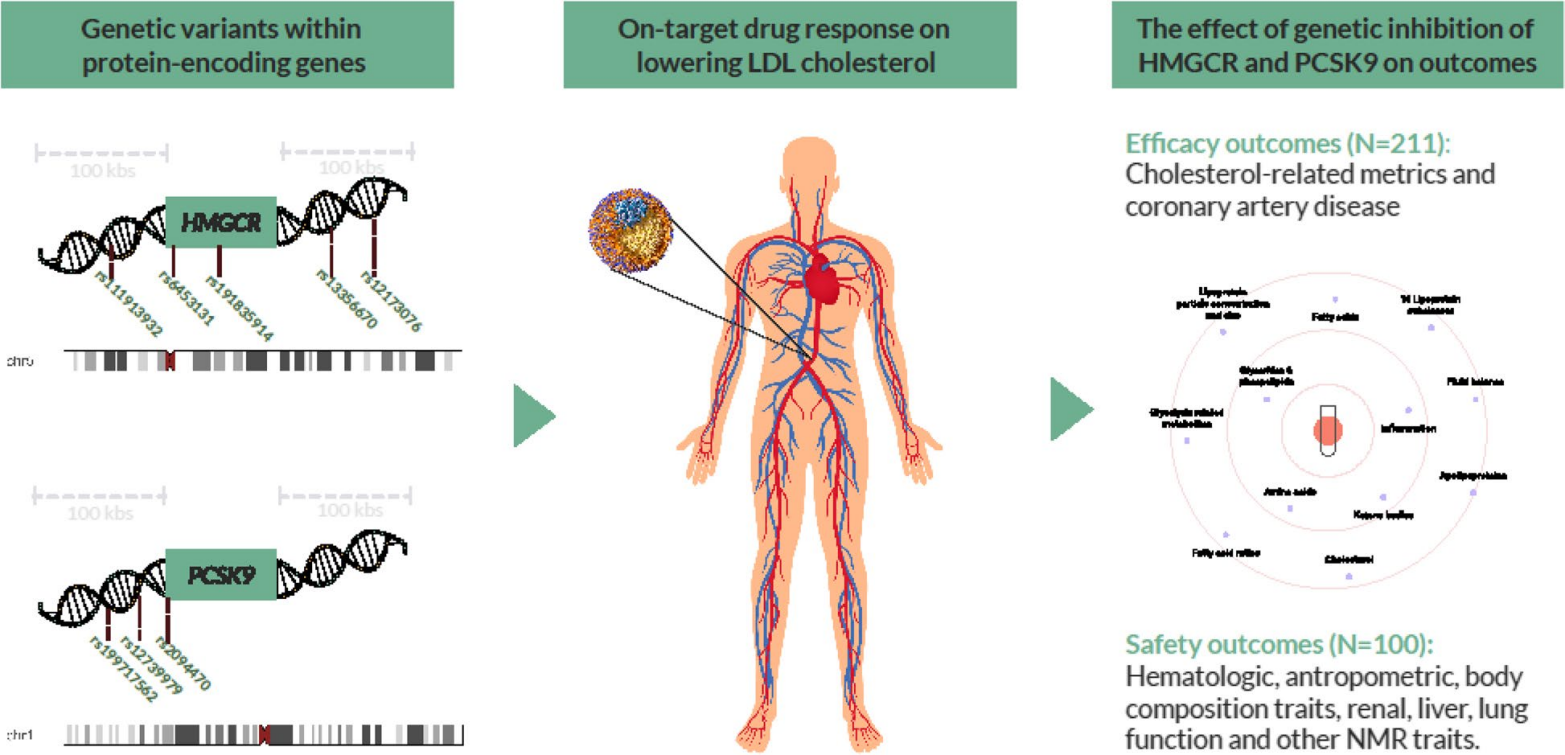
Schematic diagram of drug target Mendelian randomization study design

This is a drug-target Mendelian randomization study using individual and summary data, which relies on three instrumental variable assumptions. First to satisfy relevance, genetic variants within the protein-encoding genes (*HMGCR*, *PCSK9*, and *NPC1L1*) and associated with LDL-C were used to proxy the use of statins, PCSK9 inhibitors, and ezetimibe respectively. Second, the use of genetic variants not associated with confounders of exposure-outcome association addresses independence. Third, genetic variants are independent of the outcome given the exposure thereby addressing exclusion restriction [11]. The schematic diagram of the study design is depicted in Fig. 1. This study is reported according to Strengthening the reporting of observational studies in epidemiology using Mendelian randomization (STROBE-MR) statement (https://www.strobemr.org/).

### “Children of 1997” birth cohort

The Hong Kong “Children of 1997” birth cohort is a population-representative Chinese birth cohort, which recruited 88% of all ethnic Chinese births (*n* = 8327) in Hong Kong in April and May 1997 [12]. Recruitment was conducted at all the Maternal and Children Health Centres in Hong Kong at their first postnatal visit for free preventive care and immunizations. Infant and family characteristics were ascertained using a self-administered questionnaire [13]. In 2005, record linkage was established to obtain routine information and clinical measurements (96% successful matching, *n* = 7999). During the Biobank clinical follow-up phase 1 in 2013–2016, at 17.5 years (*n* = 3460), with a supplementary Biobank clinical follow-up phase 2 in 2017 (*n* = 158), participants provided biospecimen (blood, saliva, urine, stool, hair, and nails) and completed comprehensive measurements (e.g., anthropometrics and lung function). Fasting blood samples and their derivatives (e.g., buffy coat and plasma) were used for biochemical assays such as liver function tests and metabolomic profiling using high-throughput nuclear magnetic resonance (NMR) spectroscopy metabolomics (Nightingale Health Ltd., Helsinki, Finland, https://nightingalehealth.com/) [14], which assessed routine lipids, 14 lipoprotein subclasses, esterified fatty acid composition, and a broad range of low-molecular-weight metabolites, such as amino acids, ketone bodies, and glycolysis and gluconeogenesis-related metabolites [14, 15].

DNA extraction from either blood, buffy coat, or saliva samples, where appropriate, and genotyping were performed for 3582 participants by the Centre for PanorOmic Sciences, The University of Hong Kong, with Infinium Asian Screening Array BeadChip Kit (v1.0). Phasing was performed using Eagle v2.4, and genotyping imputation was performed with 1000 Genomes phase 3 (Version 5, GRCh37/hg19) in the Michigan Imputation Server using Minimac4 software (https://imputationserver.sph.umich.edu/index.html#!). For quality control, samples with call rate < 0.98, recorded sex mismatched with genetic-inferred sex, second degree of relatedness or above, high heterozygosity (deviation > 3 standard deviation (SD)), or variants with call rate < 0.98, and imputation score < 0.3 were excluded.

### Instrument selection

We identified genetic instruments to proxy the effects of each lipid-modifying target using the Global Lipids Genetics Consortium (GLGC), the latest genome-wide association study (GWAS), which included 146,492 middle-aged (mean aged of 55.8 years) participants of East Asian (EAS) ancestry [16]. LDL-C was measured or calculated using the Friedewald equation from either fasting or non-fasting blood samples. LDL-C was corrected by dividing the LDL-C by 0.7 if a person was taking lipid-modifying medications [16]. To reduce the likelihood of false positives, we only considered genetic variants located within 100 kilobase (kb) pairs on either side of the protein-encoding genes (*HMGCR*, *PCSK9*, and *NPC1L1*), and those associated with LDL-C at genome-wide significance (*p*-value < 5 × 10^−8^) in GLGC were considered. None of the genetic variants for *NPC1L1* passed through the selection process and hence was not considered further. A recent study found that genetic variants of LDL-C identified in middle-aged adults of EAS ancestry are highly predictive of LDL-C measurement at different stages of the life-course including adolescents [17]. Nevertheless, we cross-verified the variant-LDL-C associations in the “Children of 1997” birth cohort, adjusted for age at sample collection, sex, and the top 6 principal components (PC) of ancestry. We selected variants associated with LDL-C in two independent cohorts (GLGC and “Children of 1997” birth cohort, *p*-value < 0.05) and in low linkage disequilibrium (LD) (clumping *r*^2^ < 0.3, clumping window of 10,000 kb) using a reference panel of EAS ancestry from 1000 Genomes Project (phase 3). To assess the robustness of the results, we also used a single index variant (the smallest *p*-value).

### Genetic risk scores for HMGCR and PCSK9 in the “Children of 1997” birth cohort

To maximize statistical power, [18] an externally weighted genetic risk score (GRS) for HMGCR and PCSK9 was constructed for each participant in the “Children of 1997” birth cohort. Specifically, the GRS for HMGCR and PCSK9 were constructed by summing the number of all LDL-C lowering alleles for each variant in the *HMGCR* and *PCSK9* gene regions, weighted by the effect of that variant on LDL-C in EAS ancestry reported by GLGC [16].

### Positive control outcomes

To verify the validity of the GRS used to proxy the effect of HMGCR and PCSK9 inhibition, we considered cholesterol-related metrics and coronary artery disease (CAD) as the positive control outcomes, as lowering cholesterol levels and reducing CAD risk are well-established effects of HMGCR and PCSK9 inhibition [19]. We used cholesterol-related metrics from the NMR metabolomic panel of the “Children of 1997” birth cohort, including cholesterol, triglycerides, apolipoproteins, phospholipids, cholesteryl esters, free cholesterol, total lipids, other lipids, lipoprotein particle sizes, lipoprotein particle concentrations, lipoprotein subclasses, and relative lipoprotein lipid concentrations at 17.6 years which were used as efficacy outcomes (Additional file 1: Table S1). Summary genetic associations with CAD (29,319 cases and 183,134 controls) were obtained from Biobank Japan in middle-aged people of EAS ancestry [20].

### Safety outcomes

To systematically evaluate the safety of genetic inhibition of HMGCR and PCSK9, we considered extensive health outcomes assessed at ~ 17.6 years during the Biobank clinical follow-up. We included 20 hematologic traits, 12 anthropometric traits, 18 body composition traits, renal function (urea), liver function (alkaline phosphatase (ALP), alanine transaminase (ALT), bilirubin and total protein), and lung function (forced expiratory volume in the first second (FEV_1_), forced vital capacity (FVC), FEV_1_/FVC, peak expiratory flow (PEF), the mid-forced expiratory flow at 25–75% of the pulmonary volume (FEF 25–75%)). We also included other metrics from NMR metabolomic panel, i.e., amino acids, fatty acids, fluid balance, glycolysis-related metabolites, inflammation, and ketone bodies as safety outcomes given some of these metabolites (e.g., branched chain amino acid) may be related to cardiometabolic diseases [21, 22] (Additional file 1: Table S2).

### Statistical analysis

Instrument strength was assessed using approximated *F* statistics $\left(\frac{\mathrm{Beta}^2}{\mathrm{Standarderror}^2}\right)$, where a higher *F* statistic suggests weak instrument bias is less likely. Each continuous outcome was standardized (mean 0, SD 1) to facilitate comparison. With individual level “Children of 1997” birth cohort data, the associations of the HMGCR and PCSK9 GRS with these outcomes were obtained using a multivariable linear regression model adjusted for age at sample collection, sex, and the top 6 PCs of ancestry. The variance explained by the HMGCR and PCSK9 GRS were assessed as the proportion of variance remaining after covariates fitted in a linear model [23]. The effects of genetic inhibition of HMGCR and PCSK9 on the outcomes were assessed using the Wald ratio $\left(\frac{\mathrm{GRSonoutcome}}{\mathrm{GRSonLDL}-\mathrm C}\right)$. Effect sizes and 95% confidence intervals (95% CI) were presented per SD change in the outcome per SD decrease LDL-C induced by genetic inhibition of HMGCR and PCSK9. A combination of multiple imputation and inverse probability of participation/attribution weighting was used to account for potential attrition [24]. Briefly, multiple imputation based on a flexible additive regression model with predictive mean matching was used to impute the missing data for variables related to response [25]. Inverse probability weights were then obtained using a response model so as to minimize the probability of selection bias from attrition. Estimates from the analysis model were pooled from 50 computed datasets using Rubin’s rules [24]. A complete case analysis was used as the sensitivity analysis.

With summarized data from Biobank Japan, the effects of genetic inhibition of HMGCR and PCSK9 on the positive control outcome CAD were assessed using weighted generalized linear regression, i.e., a modified version of inverse-variance weighted (IVW) method that accounts for pairwise correlation between variants [26]. We obtained the correlation matrix between variants in participants of EAS ancestry using the 1000 Genomes Project (phase 3) and assessed heterogeneity from Cochran’s *Q* statistic [27].

To correct for the multiple testing of these biologically correlated outcomes, we used PC analysis to obtain the number of PC that explained 99% of the variance in the cholesterol related and safety outcomes. The *p* value threshold after correction for multiple testing was set as 6 × 10^−4^ (*α* = 0.05/75). Post hoc power calculation using type I error *α* of 0.05 and power of 0.8 was performed using an online tool (https://shiny.cnsgenomics.com/mRnd/). Data analyses and visualization were obtained using the R packages “*ggforestplot*” and “*EpiViz*,” R version 4.0.3 (R Foundation for Statistical Computing, Vienna, Austria).

## Results

### Study population

There were 3443 participants (1760 males and 1683 females) with valid genotype information and outcomes. Characteristics of these participants by tertile of HMGCR score and PCSK9 score are shown in Table 1. Compared with participants with the first tertile of HMGCR score and PCSK9 score, participants in the 3rd tertile had higher lipids.

**Table 1:** Characteristics of participants in “Children of 1997” birth cohort Biobank follow-up

		**HMGCR genetic risk score**				**PCSK9 genetic risk score**			
**Variable**	***N***	HMGCR tertile 1, *N* = 1148^a^	HMGCR tertile 2, *N* = 1148^a^	HMGCR tertile 3, *N* = 1147^a^	*p*-value^b^	PCSK9 tertile 1, *N* = 1148^a^	PCSK9 tertile 2, *N* = 1148^a^	PCSK9 tertile 3, *N* = 1147^a^	*p*-value^b^
Age	3443	17.48 (0.72)	17.72 (0.86)	17.61 (0.81)	< 0.001	17.46 (0.78)	17.25 (0.27)	18.11 (0.92)	< 0.001
Sex	3443				0.06				0.19
Female		536/1148 (47%)	592/1148 (52%)	555/1147 (48%)		549/1148 (48%)	548/1148 (48%)	586/1147 (51%)	
Male		612/1148 (53%)	556/1148 (48%)	592/1147 (52%)		599/1148 (52%)	600/1148 (52%)	561/1147 (49%)	
SBP (mmHg)	3433	116.04 (11.91)	115.46 (12.66)	115.44 (12.24)	0.38	116.10 (12.16)	115.34 (11.95)	115.50 (12.69)	0.37
DBP (mmHg)	3433	69.61 (8.05)	69.59 (8.30)	69.74 (8.09)	0.79	69.69 (7.99)	69.54 (8.11)	69.70 (8.34)	0.74
BMI (kg/m^2^)	3438	20.91 (3.36)	20.85 (3.45)	20.98 (3.75)	0.7	20.96 (3.56)	20.86 (3.38)	20.92 (3.63)	0.84
Total cholesterol (mmol/L)	3342	4.22 (0.72)	4.32 (0.72)	4.36 (0.70)	0.001	4.24 (0.70)	4.25 (0.70)	4.41 (0.72)	< 0.001
LDL-C (mmol/L)	3342	1.58 (0.36)	1.61 (0.35)	1.64 (0.35)	< 0.001	1.58 (0.35)	1.59 (0.35)	1.67 (0.36)	< 0.001
Triglycerides (mmol/L)	3342	0.88 (0.34)	0.87 (0.33)	0.92 (0.40)	0.039	0.89 (0.35)	0.88 (0.36)	0.91 (0.37)	0.24
Apolipoprotein B (mmol/L)	3342	0.72 (0.15)	0.74 (0.15)	0.75 (0.15)	< 0.001	0.73 (0.14)	0.73 (0.15)	0.76 (0.15)	< 0.001

^a^Mean (SD); *n*/*N* (%)

^b^Kruskal-Wallis rank sum test; Pearson’s chi-squared test

### The validity of genetic inhibition of HMGCR and PCSK9

In total, 5 variants (rs111913932, rs6453131, rs191835914, rs13356670, and rs12173076) were selected for targeting HMGCR and 3 variants (rs12739979, rs199717562, and rs2094470) for PCSK9. Their associations with LDL-C in GLGC and “Children of 1997” are summarized in Additional file 1: Table S3. There was little evidence of weak instrumental bias as the *F*-statistics were greater than 10 (HMGCR: 38 to 266 and PCSK9: 34 to 42). HMGCR and PCSK9 GRS explained 0.54% and 0.27% of LDL-C, and thus, our study was adequately powered to detect an odds ratio of 0.77 and 0.67 per SD reduction in LDL-C for CAD and a beta coefficient of ± 0.46 and ± 0.60 per SD reduction in LDL-C for the continuous outcomes. Genetic inhibition of each lipid-modifying target was associated with lower CAD risk (HMGCR: 0.66 per 1 SD reduction in LDL-C, 95% CI 0.54 to 0.81 and PCSK9: 0.55, 95% CI 0.34 to 0.90). Suggestive evidence of heterogeneity was found for the effects of genetic inhibition of HMGCR on CAD, which may be due to the missense variant of rs19183514 (Additional file 1: Tables S3-S4).

### The effect of genetic inhibition of HMGCR and PCSK9 on efficacy outcomes

**Fig. 2:**
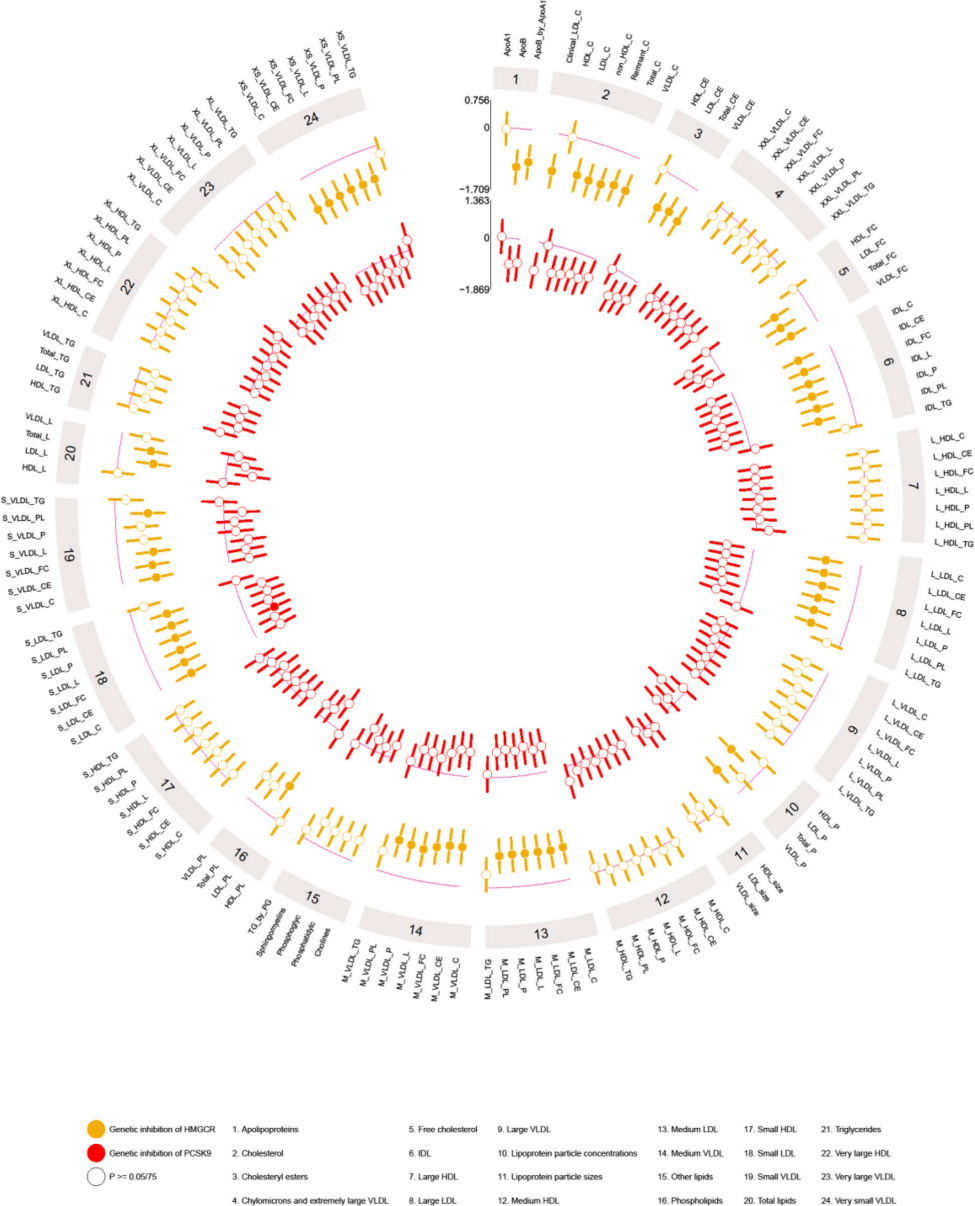
Genetic inhibition of HMGCR and PCSK9 (per lower LDL-C in SD) on efficacy outcomes in “Children of 1997” birth cohort Biobank follow-up

Genetic inhibition of HMGCR was associated with substantial reduction in several measures of cholesterol (total cholesterol, non-high density lipoprotein cholesterol (non-HDL-C), remnant cholesterol, very low lipid density lipoprotein (VLDL-C), clinical LDL-C and LDL-C) and apolipoproteins (ApoB and the ratio of apolipoprotein B to apolipoprotein A1 (ApoB/ApoA1)); *p*-value ≤ 6 × 10^−4^ (Fig. 2 and Additional file 1: Table S5). In addition, genetic inhibition of HMGCR was also associated with lower lipoprotein subclasses, including cholesterol (-C), cholesterol esters (-CE), free cholesterol (-FC), total lipids (-L), particle concentration (-P), and phospholipids (-PL) in intermediate-density lipoprotein (IDL), large LDL (L-LDL), median LDL (M-LDL), medium VLDL (M-VLDL), small LDL (S-LDL), small VLDL (S-VLDL), and very small VLDL (XS-VLDL). The metabolic profiling of genetic inhibition of PCSK9 was similar to genetic inhibition of HMGCR, for example, genetic inhibition of HMGCR and PCSK9 led to reduction of ApoB (HMGCR beta: − 1.06 SD, − 1.52 to − 0.60, *p*-value = 5 × 10^−6^ and PCSK9 beta: − 0.93 SD, − 1.56 to − 0.31, *p*-value = 3 × 10^−3^) (Fig. 2 and Additional file 1: Table S5). Similar association of genetic inhibition of HMGCR and PCSK9 and efficacy outcomes were found in sensitivity analyses using complete case and index variant analyses (Additional file 1: Table S6-7).

### The effect of genetic inhibition of HMGCR and PCSK9 on safety outcomes

**Fig. 3:**
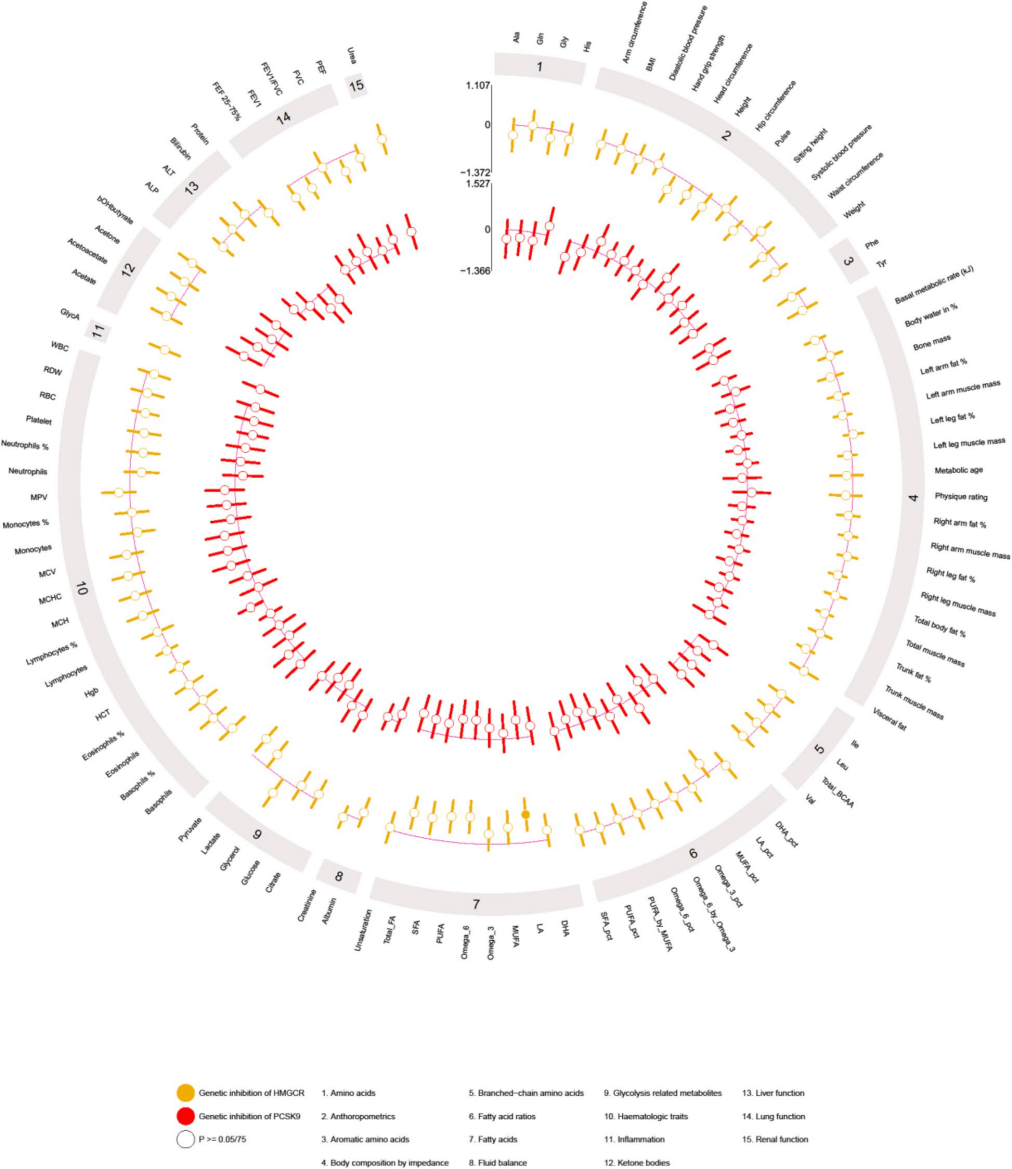
Genetic inhibition of HMGCR and PCSK9 (per lower LDL-C in SD) on safety outcomes in “Children of 1997” birth cohort Biobank follow-up

Genetic inhibition of HMGCR was associated with lower linoleic acid (LA) (beta: − 0.79 SD, 95% CI − 1.25 to − 0.34, *p*-value = 6 × 10^−4^). In addition, genetic inhibition of HMGCR was nominally associated with lower omega-6, polyunsaturated, total, saturated, and monounsaturated fatty acids, pyruvate, FEV1, FVC, head circumference, height, and higher glycerol (Fig. 3 and Additional file 1: Table S8). There was no association of genetic inhibition of HMGCR with other fatty acids or their ratios, amino acids, aromatic amino acids, branched-chain amino acids, fluid balance, glycolysis-related metabolites, inflammation, ketone bodies, anthropometric traits, body composition, hematological traits, markers of liver function, lung function, or renal function. Similarly, no association of genetic inhibition of PCSK9 with these safety outcomes was observed (Fig. 3 and Additional file 1: Table S8). Sensitivity analyses from complete case analyses and index variant analyses gave similar conclusions (Additional file 1: Table S9-10).

## Discussion

This is one of the first drug-target Mendelian randomization studies to assess the efficacy and safety of HMGCR inhibitors and PCSK9 inhibitors among Chinese adolescents*.* We found metabolic profiling was similar for genetic mimics of HMGCR inhibitors and PCSK9 inhibitors, and their efficacy in lowering apolipoproteins and cholesterol were similar to previous RCTs and Mendelian randomization studies in European and EAS adults [28–31]. A systematic review of RCTs showed relatively short-term safety of statins (no more than 48- week) and PCSK9 inhibitors (24-week) in pediatric patients with FH [32, 33]. Our study extends by providing genetic evidence that longer-term on-target effect of statins and PCSK9 inhibitors did not appear to have clear adverse effects on a wide range of health outcomes in the general Chinese adolescents.

Selecting the appropriate genetic instruments to proxy effects of medication has been challenging and not as standardized as compared to conventional Mendelian randomization studies [34]. Nevertheless, our findings that both genetic inhibition of HMGCR and PCSK9 was associated with lower risk of CAD is consistent with Mendelian randomization studies in EAS and Europeans [19, 31, 35] and with RCTs [36], and so provides some validation. Consistent with earlier RCTs in children and adolescents with FH, [37–39] we did not find an association of genetic inhibition of HMGCR with liver function, possibly because of lack of statistical power. The infrequent reported statin-associated asymptomatic increase in transaminases (> 3 times the upper limit of normal) and hepatotoxicity reported in trials are likely due to idiosyncratic or immune allergic reactions [40]. However, in 2012, the US Food and Drug Administration recommended removal of routine periodic monitoring of liver enzymes among statins users [41].

Majority of the trials on safety among adolescents were restricted to those with FH. However, with increasing rates of sedentary behaviors and obesity, it is likely that statins and/or PCSK9 inhibitors would be potential pharmaceutical interventions for the overall pediatric population since the safety profiles of these interventions are not well documented in long-term studies [42]. Using genetics, this study did not provide strong evidence for possible adverse effects regarding the use of these medications. More importantly, this study provides a proof of concept in using genetics to infer possible side effects of medications in adolescents where large-scale trials with long duration are more difficult to conduct [43]. With increasing global trends in other cardiometabolic risk factors such as hypertension and hyperglycemia among adolescents [44, 45], drug-target Mendelian randomization leverages genetics, and appropriate surrogate and safety endpoints in adolescents provide a potentially more cost-effective approach to identify adverse effects prior to the conduct of observational studies and RCTs. Establishing possible safety profile can also help inform corresponding clinical management [6–9].

This drug-target Mendelian randomization study has some limitations. First, the study used genetic variants to mimic the on-target effect of life-long (including developmental) exposure to a relatively modest LDL-C reduction induced by genetic inhibition of HMGCR and PCSK9 among Chinese adolescents. This may not be equivalent to the effects of pharmacological interventions administered at specific time points, higher dosages, for shorter periods, or to individuals with particular indications such as FH [46]. Second, we cannot assert that the observed associations in this study are solely attributable to the LDL-C reduction properties of the medication. For example, previous studies have suggested that statins can have pleiotropic effects related to cardiovascular outcomes [47, 48]. A head-to-head comparison with a conventional multivariable Mendelian randomization study of LDL-C, while adjusting for other lipid traits, could help address these research gaps. Third, although we have included rare to common variants to mimic HMGCR and PCSK9 inhibition, a recent study suggests that the inclusion of rare and loss-of-function (LoF) variant may provide further insights into the efficacy and safety of therapeutic targets [49]. However, LoF variants tend to maintain at very low frequencies in populations, [50] and studies exploring the health effects of LoF variants typically require extensive biobanks, such as the UK Biobank*.* Fourth, genetic variants for genetic inhibition of *HMGCR* and *PCSK9* were selected and validated in people of EAS descent only. While there is a minor imbalance in age distribution across tertiles of HMGCR and PCSK9 scores, age was adjusted for in the study, making it unlikely to explain our findings. We were also precluded from using colocalization to assess the suitability of the chosen instruments, because of the lack of relevant expression or protein quantitative trait loci data in EAS. Nevertheless, the variants used for statins and PCSK9 inhibitors are quite highly correlated with those previously used in Europeans and EAS [31]. The diversity in human genetics highlights the need for ethnicity-specific GWAS and corresponding Mendelian randomization studies, with appropriate controls for population stratification, to generate contextually specific evidence [51]. It would be valuable to replicate this study in the adolescents of other ethnicities. Fifth, constrained by sample size and hence statistical power, we cannot rule out the possibility of false negatives. Similarly, due to statistical power issues, we were unable to consider dichotomized outcomes (e.g., hypertension and prediabetes) or sex-specific associations [52]. Furthermore, statistical power may reduce the capability to detect false negatives when accounting for multiple corrections. In this context, results should be interpreted with caution, as any nominally significant associations (e.g., lung function and height) or associations with large effect sizes (e.g., acetate) may suggest potential safety concerns and require additional replications in larger studies [53]. Further research to validate specific side effects could facilitate a more targeted approach that circumvents the challenges of multiple testing when corresponding birth cohort studies are not extensive. Sixth, although we have included a wide range of health outcomes (*n* = 100), such as metabolomics, we did not cover all possible side effects, including muscle symptoms and related biomarkers (e.g., creatine kinase and myoglobin) [54] and testosterone reduction [55]. Lastly, our study is more likely to detect milder side effects, as lethal variants may have led to reduced participation in GLGC (i.e., selection bias) due to the presence of more severe side effects and developmental disorders. This could, in turn, impact the likelihood of identifying and including these variants in our study. A broader spectrum of potential side effects should be assessed in larger studies, such as linking electronic health records of clinical outcomes to biobank studies, e.g., the All of Us study, which recruited over 40,000 participants aged 18–29, [56] and utilizing birth cohorts with whole genome sequencing data.

## Conclusions

This study did not find evidence that raise substantial concerns about the longer-term safety of statins and PCSK9 inhibitor in this Chinese adolescent population; these findings require verification in larger studies. Our study serves as a proof-of-concept study showing the use of well-characterized birth cohorts with multi-omics data (genetics and metabolomics) and phenotypes to facilitate the assessment of the efficacy and safety of drug targets among adolescents.

### Supplementary Information

**Additional file 1. Table S1.** The primary efficacy outcomes from the “Children of 1997” birth cohort. **Table S2.** The safety outcomes from the “Children of 1997” birth cohort. **Table S3.** The genetic variants selected for the HMGCR score and PCSK9 score and their association in the Global Lipids Genetics Consortium and the “Children of 1997” birth cohort. **Table S4.** The effect of genetic inhibition of HMGCR and PCSK9 on coronary artery disease in East Asian. **Table S5.** The effect of genetic inhibition of HMGCR and PCSK9 on efficacy outcomes in the “Children of 1997” birth cohort. **Table S6.** The effect of genetic inhibition of HMGCR and PCSK9 on efficacy outcomes using complete case analysis in the “Children of 1997” birth cohort. **Table S7.** The effect of genetic inhibition of HMGCR and PCSK9 (using index variant) on efficacy outcomes in the “Children of 1997” birth cohort. **Table S8.** The effect of genetic inhibition of HMGCR and PCSK9 on safety outcomes in the “Children of 1997” birth cohort. **Table S9.** The effect of genetic inhibition of HMGCR and PCSK9 on safety outcomes using complete case analysis in the “Children of 1997” birth cohort. **Table S10.** The effect of genetic inhibition of HMGCR and PCSK9 (using index variant) on safety outcomes in the “Children of 1997” birth cohort. Supplementary Note. R code for Mendelian randomization analysis, principal component analysis, and visualization.

**Additional file 2. **

## Data Availability

Information on two lipid-modifying drug targets (*HMGCR* and *PCSK9*) were sourced from the druggable genome. The genome-wide association summary statistics for LDL-C, restricted to population of East Asian ancestry, was downloaded from Global Lipids Genetics Consortium (http://csg.sph.umich.edu/willer/public/glgc-lipids2021/). The genome-wide association summary statistics for coronary artery disease was downloaded from Biobank Japan (https://pheweb.jp/downloads). Data from the “Children of 1997” birth cohort are available upon reasonable request from the “Children of 1997” data access committee: aprmay97@hku.hk. R code for Mendelian randomization analysis, principal component analysis, and visualization are in Additional file 2.

## Abbreviations

ALP: Alkaline phosphatase
ALT: Alanine transaminase
ApoB: Apolipoprotein B
ApoB/ApoA1: The ratio of apolipoprotein B to apolipoprotein A1
CAD: Coronary artery disease
EAS: East Asian
FEF 25–75%: Mid-forced expiratory flow at 25–75% of the pulmonary volume
FH: Familial hyperchloremia
FEV1: Forced expiratory volume in the first second
FVC: Forced vital capacity
GLGC: Global Lipids Genetics Consortium
GRS: Genetic risk score
GWAS: Genome-wide association study
HMGCR: Three-hydroxy-3-methylglutaryl coenzyme A
IVW: Inverse-variance weighted
LD: Linkage disequilibrium
LDL-C: Low-density lipoprotein cholesterol
NMR: Nuclear magnetic resonance
Non-HDL-C: Non-high density lipoprotein cholesterol
PC: Principal components
PCSK9: Proprotein convertase subtilisin/kexin type 9
PEF: Peak expiratory flow
RCT: Randomized controlled trials
SD: Standard deviation
VLDL-C: Very low lipid density lipoprotein cholesterol
