# Supplementary material for: Assessing the safety of lipid-modifying medications among Chinese adolescents: a drug-target Mendelian randomization study
Source: BMC Med. 2023 Oct 31;21:410. doi: 10.1186/s12916-023-03115-y (PMC10617134; doi:10.1186/s12916-023-03115-y)
Supplement: Supplementary file 2 — Additional file 2. [file 12916_2023_3115_MOESM2_ESM.pdf]

R code for Mendelian randomisation analysis, principal component analysis, and visualization

The associations of the genetic variants with the exposure are denoted betaXG with SEs sebetaXG. The associations of the genetic variants with the outcome are denoted betaYG with SEs sebetaYG.

Using summary-level data, these associations were obtained from genetic consortia.

```
library (TwoSampleMR)
library (MendelianRandomization)
```

Instrument strength approximated F-statistics:

```
Fstat = (betaXG)^2 / (sebetaXG)^2
```

Harmonise the alleles and effects between the exposure and the outcome:

```
dat = harmonise_data(exposure_data, outcome_data, action=2)
dat = dat %>% filter(mr_keep==TRUE)
```

Inverse variance weighting estimates, accounting for the genetic correlation matrix. Fixed effects models are used throughout this paper because all genetic variants all from the same gene region (*HMGCR* and *PCSK9*) that should have similar mechanisms of effect:

```
dat2 = dat_to_MRInput(dat, get_correlations = TRUE, pop='EAS')
MendelianRandomization::mr_ivw(dat2[[1]], correl=TRUE, model='fixed')
```

Using individual-level data from the "Children of 1997" birth cohort, these associations were estimated using linear regression (continuous outcomes)

```
library(ggforestplot)

df_long =
  df_full_data %>%
  dplyr::select(names(exposures), names(outcomes), sex, age, paste0('PC', 1:6) %>%
  # Log-transform and scale biomarkers
  dplyr::mutate_at(
    .vars = dplyr::vars(tidyselect::all_of(names(outcomes))),
    .funs = ~ .x %>% log1p() %>% scale %>% as.numeric()) %>%
  # Collapse to a long format
  tidyr::gather(
    key = biomarkerid,
    value = biomarkervalue,
    tidyselect::all_of(names(outcomes)))
```

Estimate genetic associations, adjusted for sex, age and top six PCs:

```
assoc_per_biomarker_HMGCR =
  ggforestplot::discovery_regression(
    formula = formula(biomarkervalue ~ HMGCR+sex+age+PC1+PC2+PC3+PC4+PC5+PC6),
    key = biomarkerid, predictor = HMGCR, df_long = df_long, model = "lm")
```

Estimate the associations of X on Y using the Wald ratio:

```
betaXY = betaYG/betaXG
seXY = sebetaYG/abs(betaXG)
pval = pnorm(abs(betaXY)/seXY, lower.tail=F)*2
```

Perform principal component analysis:
```
df_pca =
  df_full_data %>%
  select(names(outcomes) %>%
  nest(data = dplyr::everything()) %>%
  mutate(
    pca = map(data, ~ stats::prcomp(.x, center = TRUE, scale = TRUE)),
    pca_aug = map2(pca, data, ~broom::augment(.x, data = .y)))
```

Estimate the amount of variance explained by each principal component"
```
df_pca_variance =
  df_pca %>%
  unnest(pca_aug) %>%
  summarize_at(.vars = vars(starts_with(".fittedPC")), .funs = ~ var(.x)) %>%
  gather(key = PC, value = variance) %>%
  mutate(cumvar = cumsum(variance / sum(variance)),
        PC = str_replace(PC, ".fitted", ""))
```

Find the number of principal components that explain 99%:
```
pc_99 =
  df_pca_variance %>%
  filter(cumvar <= 0.99) %>%
  nrow()
```

Corrected significance threshold:
```
psignif <- signif(0.05 / pc_99, 1)
```

Visualization using circus_plot from 'EpiViz' package:
```
library(EpiViz)

circos_plot(
track_number = 2,
track1_data = HMGCR, track2_data = PCSK9,
track1_type = "points", track2_type = "points",
label_column = 3,
section_column = 2,
order = F,
order_column = 5,
estimate_column = 11,
pvalue_column =16,
pvalue_adjustment = 75,
lower_ci = 13, upper_ci = 14,
legend = T,
track1_label = 'Genetic inhibition of HMGCR',
track2_label = 'Genetic inhibition of PCSK9',
pvalue_label = '<=0.0006',
circle_size = 8,
track1_height = 0.30, track2_height = 0.25,
equal_axis = T,
origin=0,
colours=c("#E41A1C", "#4DAF4A"))
```
